# Proteasome subunit-α type-6 protein is post-transcriptionally repressed by the microRNA-4490 in diabetic nephropathy

**DOI:** 10.1042/BSR20180815

**Published:** 2018-10-31

**Authors:** Ying Feng, Jin Ming-yue, Liu Dong-wei, Li Wei

**Affiliations:** 1 Department of Endocrinology, The Seventh Affiliated Hospital of Sun Yat-sen University, Shenzhen, Guangdong 518107, China; 2 Department of Traditional Chinese Medicine, The Seventh Affiliated Hospital of Sun Yat-sen University, Shenzhen, Guangdong 518107, China; 3 Department of Dermatology, Beijing Children’s Hospital, Capital Medical University, National Center for Children’s Health, Beijing 100045, China

**Keywords:** diabetic nephropathy, ESRD, microRNA-4490, Proteasome subunit alpha type-6 protein, PSMA6

## Abstract

A common complication of both type I and type II diabetes is nephropathy, characterized by accumulation of extracellular matrix in the glomerular mesangium. This indicates a central role of mesangial cells in the pathophysiology of diabetic nephropathy. Using the proteomic approach, it was earlier elucidated in a rat model that the proteasome subunit-α type-6 protein (PSMA6) is suppressed in the renal cortex in nephropathic kidney. However, the underlying mechanism effecting suppression of PSMA6 protein in the renal cortex is not yet known. Twenty diabetic patients were enrolled and the expression level of PSMA6 in them was detected by immunohistochemistry. The protein and mRNA expression levels of PSMA6 in NRK-52E cells under high glucose condition were determined by Western blot and quantitative real-time PCR, respectively. Dual luciferase assay was used to detect the relationship of PSMA6 and miR-4490. Our results show that PSMA6 protein is down-regulated in patients with diabetic nephropathy compared with healthy control. Using the NRK-52E cell line cultured under high glucose condition as an *in vitro* model of diabetic nephropathy, we show that loss of PSMA6 protein expression occured independent of changes the in *PSMA6* mRNA expression. We next elucidate that *PSMA6* mRNA is post-transcriptionally regulated by the microRNA (miRNA)-4490, whose expression is inversely correlated to PSMA6 protein expression. Using reporter assays we show that *PSMA6* is a direct target of the miR-4490. Exogenous manipulation of miR-4490 levels modulated expression of PSMA6, indicating that miR-4490 can be tested as a biomarker for nephropathy in diabetic patients.

## Introduction

A common complication of type I and type II diabetes is nephropathy, where extracellular matrix leaks into the glomerular region of the kidney [1]. This microvascular disorder occurs in 25–40% of all diabetic patients and is the biggest cause of both end stage renal disease (ESRD) and diabetic mortality [2].

ESRD or mortality from diabetic nephropathy results from multiple complications, inclusive of glomerular hypertension, oxidative stress, activation of protein kinase C, TGF-β, and accumulation of advanced glycosylation end products [3–5]. However, the underlying mechanisms contributing to the pathophysiology of diabetic nephropathy are still largely unknown.

One study had shown via microarray that approximately 200 genes are differentially expressed in the resident mesangial cells of nephropathic kidney [6]. On the other hand, proteomic studies have identified different set of differentially expressed proteins in diabetic kidney [7–10]. Using a rat model of diabetic nephropathy it was observed that expression of proteasome subunit-α type-6 protein (PSMA6) is suppressed in renal cortex of nephropathic kidney [9]. However, regulatory mechanisms underlying PSMA6 protein expression in diabetic nephropathy are not completely defined.

MicroRNAs (miRNAs) are 22 nucleotides long RNA that do not encode a protein product, but function to regulate expression of other messenger RNAs (mRNAs) [11]. Given their important role in regulating gene expression, it is not a surprise that miRNAs have been found to be involved to regulate both normal physiological as well as disease processes.

Here, using an *in vitro* model of diabetic nephropathy, NRK-52E cells maintained under high glucose conditions [12], we found that *PSMA6* gene expression is not regulated at the mRNA level, but at the post-transcriptional level. *In situ* prediction algorithm suggested that the miR-4490 might be targeting *PSMA6*. Our experimental results show that PSMA6 protein expression is indeed post-transcriptionally inhibited by miR-4490. Modulation of miR-4490 levels rescued PSMA6 protein expression in the NRK-52E cells.

## Materials and methods

### Patients

The study protocol was approved by the Seventh Affiliated Hospital, Sun Yat-sen University. Tissue specimens (diabetic nephropathy and control) were obtained from 20 Chinese patients at the Seventh Affiliated Hospital, Sun Yat-sen University between 2016 and 2017 and fixed in 10% formaldehyde.

### Cell culture

NRK-52E cells were obtained from the Chinese Center for Type Culture Collection (Shanghai, China). The cells were cultured in DMEM containing 10% FBS and penicillin/streptomycin in an incubator containing 5% CO_2_ and maintained at 37°C. Cells were initially grown in DMEM containing 0.5% FBS for 24 h (day 0), before being treated with 5.6 mM glucose (low glucose group) or 30 mM glucose (high glucose group) for indicated time periods. Where time periods are not indicated, it signifies that the cells were grown under the mentioned conditions for 72 h.

### Immunohistochemistry

Tissue samples from patients were fixed using formaldehyde and embedded in paraffin and were was cut into 5 μm sections. The sections were deparaffinized and rehydrated using xylene and ethanol, respectively. Endogenous peroxidase blocking was performed using 0.3% (v/v) hydrogen peroxide after antigen retrieval was completed in the citrate buffer. The sections were stained using anti-PSMA6 antibody (Abcam, MA, U.S.A.) overnight at 4°C using routine protocols. And then the sections were incubated with biotinylated goat anti-mouse IgG (Abcam, MA, U.S.A.) for 30 min as secondary antibodies. The sections were scored by a pathologist in a blinded fashion.

### RNA and miRNA extraction and quantitative real-time PCR

Trizol was used to isolate mRNA and miRNA from cell line NRK-52E. TaqMan miRNA assays (Thermo Fisher Scientific, Shanghai, China) were used to detect miR-4490 and the internal normalization control, *RNU6B*. TaqMan Gene Expression probes were used to quantitate transcript level of *Psma6* and the internal normalization control, *Gapdh*. The assays were performed in triplicates and done at least three different times and 2^−ΔΔC^_t_ method was used to determine the relative expression levels.

### Reporter plasmids, transfection, and assay

The *PSMA6* 3′-UTR clone was obtained from Origene. The *PSMA6* 3’-UTR mutant construct, where nucleotides 15–22 of the 3’-UTR were deleted, corresponding to the hsa-miR-4490 binding site, using site directed mutagenesis. The pGL3 firefly luciferase vector (Qiagen) was used as the internal normalization control for all the experiments. NRK-52E cells were transfected with the wild-type and mutant reporter plasmids and luciferase assays were performed 24 h after transfection. Data were presented as means (relative fluorescent units) ± S.D.

### Cell lysis and Western blot

RIPA buffer supplemented with protease and phosphatase inhibitor cocktail were used for cell lysis. Protein concentrations were determined using BCA Assay Kit (Pierce, Rockford, IL) according to manufacturer’s instructions. Then 30 μg protein was separated by SDS-polyacrylamide gel and transferred to the PVDF membrane. Blots were blocked with 5% (w/v) nonfat dry milk for 1 h and probed with primary antibodies PSMA6 (dilution: 1:1000) and GAPDH (dilution: 1:1000) (Abcam, Waltham, MA, U.S.A.). The GAPDH was used as internal control. The blots were imaged using ECL Plus Western blotting detection reagents and quantized by Image J software (National Institute of Mental Health [NIMH], Bethesda, Maryland, U.S.A.).

### Statistical analyses

SPSS version 16 (IBM Corporation, NY) was used for statistical analysis. Data were presented as mean ± S.D. Differences between two groups were assessed using Student’s *t* test. A *P*<0.05 was considered as statistically significant.

## Results

**Figure 1:**
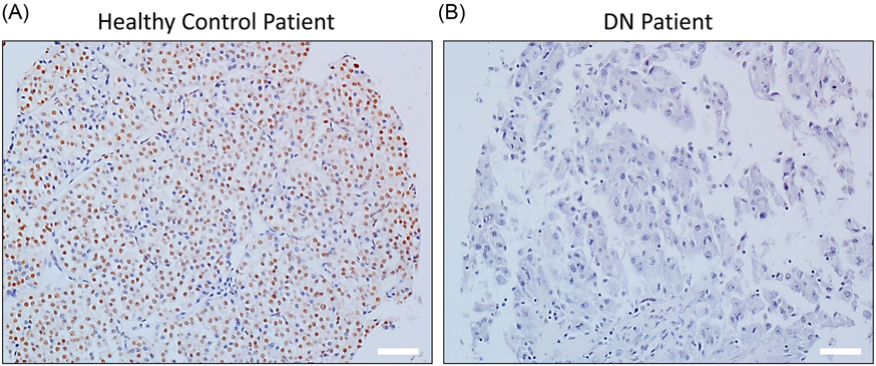
PSMA6 protein expression is down-regulated in renal tissue obtained from patients with diabetic nephropathy Representative immunohistochemistry images showing PSMA6 expression in renal tissue obtained from healthy controls (**A**) or patients with diabetic nephropathy (DN) (**B**). Scale bar – 40 µm.

It has been previously shown using a rat model of diabetic nephropathy that PSMA6 protein expression is suppressed in the renal cortex [9]. We thus initially determined PSMA6 protein expression in tissue specimens obtained from patients with diabetic nephropathy and healthy controls. Patients with diabetic nephropathy had undetectable levels of PSMA6 compared with healthy controls (Figure 1).

**Figure 2:**
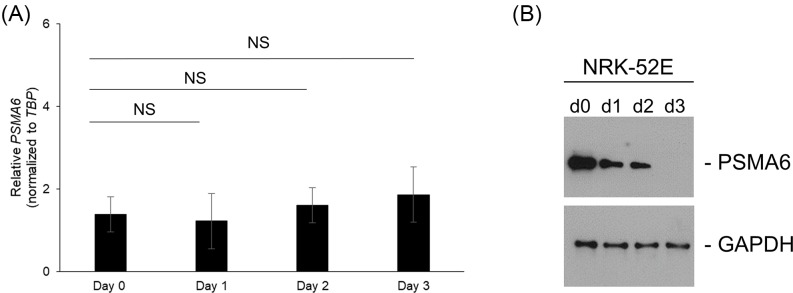
Down-regulation of PSMA1 expression in NRK-52E cells maintained under high glucose occurs independently of changes in PSMA6 mRNA levels (**A**) Relative expression of *PSMA6* mRNA in NRK-52E cells maintained in culture with high glucose over 3 days. (**B**) Relative expression of PSMA6 protein under the same conditions as (**A**). Blots were probed with GAPDH antibody to confirm equal loading across the different lanes.

To investigate what regulates PSMA6 protein expression during diabetic nephropathy, we next used the *in vitro* NRK-52E cells, which have been shown to induce diabetic nephropathy-like features inclusive of gene and protein expressions when cultured at high glucose concentrations for prolonged periods [12]. We initially determined relative expression of *PSMA6* mRNA in NRK-52E cells maintained in culture with high glucose over 3 days. *PSMA6* mRNA expression did not change significantly (Figure 2A), indicating that regulation of PSMA6 protein expression is not at the level of transcription. However, PSMA6 protein expression was decreased (Figure 2B), which was not rescued by addition of the proteasome inhibitor, MG-132 (data not shown), suggesting that the regulation of PSMA6 protein expression is at the post-transcriptional level.

**Figure 3:**
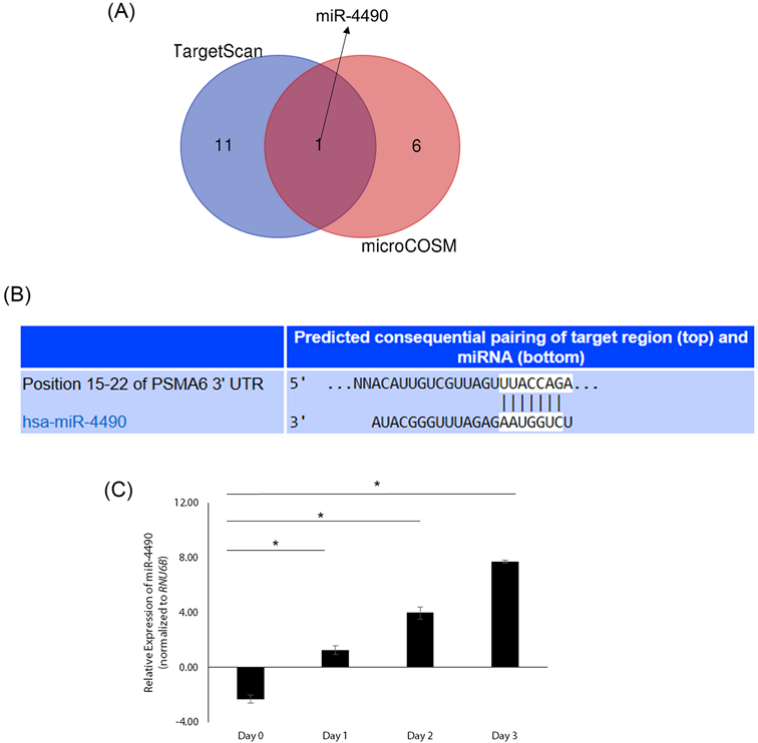
PSMA6 is a predicted target of miR-4490, whose expression varies with glucose levels (**A**) Venn diagram showing overlap of putative number of miRNAs revealed by TargetScan and MicroCosm algorithm to target *PSMA6*. MiR-4490 was the only miRNA found in both searches. (**B**) Result of TargetScan showing predicted highly conserved target site for miR-4490 in the 3’-UTR of *PSMA6* mRNA. Similar results were obtained using MicroCosm (data not shown). (**C**) Expression of miR-4490 in NRK-52E cells on day 0–3 (low glucose to high glucose condition). Data shown are changes in fold expression post-normalization to *RNU6B*.

We thus searched for putative miRNAs that might target *PSMA6* expression using TargetScan and MicroCosm algorithms. TargetScan revealed 12 putative miRNAs that could target *PSMA6* and MicroCosm revealed 7 miRNAs that could target *PSMA6* (Figure 3A). MiR-4490 was the common miRNA in both algorithms (Figure 3A,B) and was conserved across species (data not shown). Given that miRNA-mediated regulation of *PSMA6* has not been previously reported we decided to investigate it further. Given miR-4490 was the only common miRNA in both algorithms we decided to focus on miR-4490.

MiR-4490 expression significantly increased in NRK-52E cells over 3 days (Figure 3C). We next determined if miR-4490 was inhibiting translation of *PSMA6* by polysome profiling. Polysome profiling confirmed translational inhibition and mimicked the protein expression pattern (Figure 2B and data not shown).

**Figure 4:**
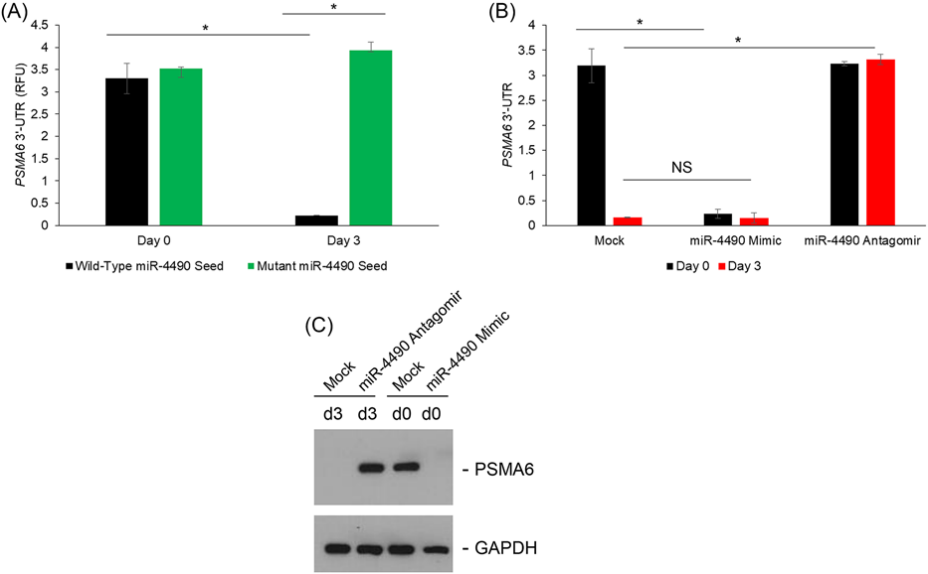
MiR-4490 targets PSMA6 and decreases its translation in the NRK-52E cells grown under high glucose (**A**) Shown are normalized luciferase expression of the wild-type and mutant *PSMA6* 3’-UTR reporter plasmid. Reporter activity of the wild-type, but not the mutant, plasmid was significantly higher on day 0 compared with day 3 for the wild-type construct (*P*<0.05). (**B**) Cells were co-transfected with the wild-type *PSMA6* luciferase reporter and either miR-4490 mimic or anti-miR-4490 antagomir. Shown are relative luciferase expression on day 0 and 3 following co-transfection with either miR-4490 mimic or anti-miR-4490 antagomir (*P<0.05). (**C**) Immunoblot analysis of PSMA6 protein expression under the same experimental conditions confirmed the results obtained in the reporter assay in (**B**).

We next wanted to confirm if *PSMA6* is truly a miR-4490 target. Luciferase reporter constructs carrying either wild-type of miR-4490 binding site mutated 3’-UTRs were used for transfecting the NRK-52E cells on day 0 (low glucose) or day 3 (high glucose) (Figure 4A). High glucose inhibited reporter activity of the wild-type plasmid (3.2 ± 0.05 folds; *P*=0.004) (Figure 4A). However, the mutant reporter activity did not decrease under high glucose conditions (Figure 4A), suggesting that the observed effects were due to miR-4490-mediated targeting of *PSMA6*.

To confirm the targeting of *PSMA6* by miR-4490, we next co-transfected miR-4490 mimic into NRK-52E cells growing under low glucose (day 0). MiR-4490 mimic inhibited reporter activity even on day 0 (Figure 4B). Conversely, co-transfection of an anti-miR-4490 antagomir into NRK-52E cells growing under high glucose (day 3) rescued the reporter activity (Figure 4B), confirming that *PSMA6* is a target of miR-4490 in this context. Our results showed that *PSMA6* is translationally silenced by miR-4490 targeting under high glucose conditions in the NRK-52E cells (Figure 4C).

## Discussion

Our results show that miRNA-4490 is up-regulated in an *in vitro* model of diabetic nephropathy, NRK-52E cells cultured under high glucose for a prolonged period, and targets *PSMA6* for post-transcriptional silencing. MiR-4490 expression thus might serve as a biomarker to predict risk of diabetic nephropathy in diabetic patients. How miR-4490 expression varies and correlates to disease progression in type I and type II diabetes patients, untreated and treated, remains to be determined in future studies.

MiRNAs have been shown to render both renal protective effects as well as involved in the pathogenesis of diabetic nephropathy [13,14]. Targeting of the zinc finger E-box binding homeobox 1/2 (*ZEB1/2*) by miR-192 has been shown to result in renal fibrosis by activation of the TGF-β signaling pathway [15]. Similarly, miR-21 has been shown to result in constitutive activation of Akt signaling pathway by targeting phosphate and tensin homolog, ultimately leading to hypertrophy and renal fibrosis [16].

The down-regulated miRNAs, inclusive of miR-29, miR-141, and miR-200, seem to be rendering renal protective effect by reducing expression of TGF-β2, COL1, COL4, and NADPH oxidase subunit 4 all factors involved in the pathophysiology of diabetic nephropathy [13,14,17–19]. The *PSMA6* gene has been shown to harbor single nucleotide polymorphism, including a 5’-UTR 8 C/G, associated with diabetes, and that *PSMA6* polymorphism might be a protective factor for ESRD [20,21]. Our results show that exogenous manipulation of miR-4490 levels can modulate expression of PSMA6, indicating that miR-4490 can be tested as a potential biomarker for nephropathy in diabetic patients.

It will be important to determine if multiple miRNA expression can provide higher prognostic value in predicting risk of diabetic nephropathy. In addition, it will also be important to estimate what regulates differential miRNA expression and how their cumulative interplay is resulting in diabetic nephropathy.

## Abbreviations

ESRD: end stage renal disease
miRNA: microRNA
mRNA: messenger RNA
PSMA6: proteasome subunit-α type-6 protein
*PTEN*: phosphate and tensin homolog
SD: standard deviation
SDS: sodium dodecyl sulfate
*ZEB1/2*: zinc finger E-box binding homeobox 1/2
