# Correction: Proteasome subunit-α type-6 protein is post-transcriptionally repressed by the microRNA-4490 in diabetic nephropathy

**DOI:** 10.1042/BSR-20180815_COR

**Published:** 2019-01-11

**Authors:** Ying Feng, Ming-yue Jin, Dong-wei Liu, Li Wei

***Bioscience Reports* (2018) 38, BSR20180815; https://doi.org/10.1042/BSR20180815**

The forenames and surnames of the second and third authors were transposed in the published version of this article. The correct names, Ming-yue Jin and Dong-wei Liu, are presented here. The online version was corrected on 20 December 2018.

